# Origami Metawall: Mechanically Controlled Absorption and Deflection of Light

**DOI:** 10.1002/advs.201901434

**Published:** 2019-10-29

**Authors:** Min Li, Lian Shen, Liqiao Jing, Su Xu, Bin Zheng, Xiao Lin, Yihao Yang, Zuojia Wang, Hongsheng Chen

**Affiliations:** ^1^ Interdisciplinary Center for Quantum Information State Key Laboratory of Modern Optical Instrumentation College of Information Science and Electronic Engineering Zhejiang University Hangzhou 310027 China; ^2^ Key Laboratory of Advanced Micro/Nano Electronic Devices & Smart Systems of Zhejiang The Electromagnetics Academy at Zhejiang University Zhejiang University Hangzhou 310027 China; ^3^ Department of Physics Massachusetts Institute of Technology Cambridge MA 02139 USA; ^4^ State Key Laboratory of Integrated Optoelectronics College of Electronic Science and Engineering Jilin University 2699 Qianjin Street Changchun 130012 China; ^5^ Division of Physics and Applied Physics School of Physical and Mathematical Sciences Nanyang Technological University 21 Nanyang Link Singapore 637371 Singapore; ^6^ School of Information Science and Engineering Shandong University Qingdao 266237 China

**Keywords:** metamaterials, optical transition, origami, reconfigurable

## Abstract

Metamaterials/metasurfaces, which have subwavelength resonating unit cells (i.e., meta‐atoms), can enable unprecedented control over the flow of light. Despite their significant progress, achieving dynamical control of both energy and momentum of light remains a challenge. Here, a mechanically tunable metawall capable of either absorbing light energy or modulating light momentum, by incorporating the magnetic meta‐atoms into a 3D printed origami grating, is theoretically designed and experimentally realized. Through mechanical stretching or compressing of the Miura‐ori pattern, the function of metawall can transit from an absorber, a mirror, to a negative reflector. Particularly, the continuously geometric deformation of the Miura‐ori lattice is a promising approach to compensate the angular dispersion in gradient metasurfaces. Considering the prominent mechanical properties and strong deformation abilities of origami structures, the findings may open an alternative avenue toward lightweight and deployable metadevices with diversified and continuously alterable electromagnetic properties.

## Graphical abstract

Here, an origami metawall capable of either absorbing light energy or modulating light momentum is proposed. With external mechanical stimuli, the functions of metawall can be switched among an absorber, a reflective mirror or a negative reflector. The findings may open an alternative avenue toward lightweight and deployable metadevices with diversified and continuously alterable electromagnetic properties.

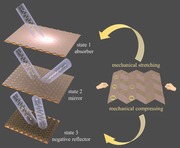

Light can be absorbed or scattered by objects, resulting in energy and momentum transfer between light and the objects. Over the past decade, metamaterials and metasurfaces^1^, ^2^, ^3^, ^4^, ^5^, ^6^, ^7^, ^8^, ^9^, ^10^ have attracted intense interests from the photonic community due to their unprecedented abilities of controlling the properties of light, including amplitude, phase and polarization. Among the numerous research branches of modulating the light energy, metamaterial absorbers^11^, ^12^, ^13^ trap the incident light and transfer their energies to phonons^14^ or electrons,^15^ providing wide applications^16^ in sensors, thermal imaging, and so on. Gradient metasurfaces are capable of manipulating the momentum of the scattered light, leading to nontrivial behaviors including anomalous refraction/reflection,^17^, ^18^, ^19^, ^20^, ^21^, ^22^, ^23^, ^24^ retroreflection,^25^, ^26^ beaming splitting^27^, ^28^, ^29^, ^30^ and focusing.^6^, ^31^ Furthermore, to achieve dynamical control over the absorption and momentum transfer of light, some approaches have been proposed, for instance, tuning absorption frequency via tunable lumped capacitors,^32^ absorption efficiency controlled by mechanical compression^33^ or external voltage,^34^ reconfigurable momentum manipulation of light utilizing liquid metal,^26^ phase‐change^35^, ^36^ and shape‐change materials.^37^ Despite so many strategies proposed to dynamically control light energy and momentum, achieving both light absorption and momentum manipulation remains a challenge due to the requirements of extreme distinct interactions between incident light and meta‐atoms. Notably, among all the proposed methods, metadevices based on controllable shape‐change materials have great potentials to achieve reversible high‐contrast optical properties. The reason is that the transition from 2D structures to 3D counterparts may yield dramatic changes in optical responses, for instance, attaining chiral responses via deformation of origami and kirigami metamaterials.^38^, ^39^ Origami/kirigami, the ancient art of folding a sheet of paper into 3D decorative shapes, is not only an inspiring technique to create sophisticated shapes but also a surprisingly versatile platform to investigate a host of shape changing metadevices with customized functionalities.^38^, ^39^, ^40^, ^41^, ^42^, ^43^ Particularly, the continuous transformation of origami/kirigami promises an unprecedented opportunity for ultrawide dynamic tuning range in reconfigurable metadevices. In our previous work,^38^ split‐ring resonators are directly printed on the polyimide film to construct reconfigurable origami metamaterial. However, folded polyimide substrate suffers from the inherent limitations on flexibly switching the folding state and the whole structure is rigid and seems “locked” at current folding state.

Here, by incorporating the magnetic meta‐atoms (copper split‐ring resonators) into a 3D printed origami grating (see more details in *Device Fabrication*), we propose and demonstrate an origami metawall which manipulates the incident light in diversified manners entirely determined by its geometric deformation (**Figure** **1**). The thickness of the 3D printed substrate at the creases are smaller than other places of the parallelogram, such configuration is beyond the limits of polyimide film based origami metamaterials for flexible shape transformation. With external mechanical stimuli, the metawall could be stretched or compressed continuously and flexibly, leading to tunable control over the energy and momentum transfer of incident light. The incident light are largely trapped (i.e., absorption) on the surface of the 2D planar metawall (state 1), while the folded metawall modifies the in‐plane momentum (*P*_∥_) of incident light. The momentum change is determined by the folding state of metawall, i.e., a slight deformation (state 2) does not change the incident momentum ($P_{∥}^{i}$) while a large deformation (state 3) provides a reversed momentum (*P^m^*) to the incident one and results in negative reflection. The optical transition can be realized via continuously tuning the folding state of the metawall. A proof‐of‐principle metawall has been fabricated in the microwave region and its performance has been validated experimentally. Considering the scalability of the Origami and its suitability for engineering deployable or foldable structures,^44^ the demonstrated concept could be extended to shorter wavelengths such as terahertz frequencies, and our design may serve as a promising platform toward lightweight and foldable photonic metadevices with diversified electromagnetic properties.

**Figure 1:**
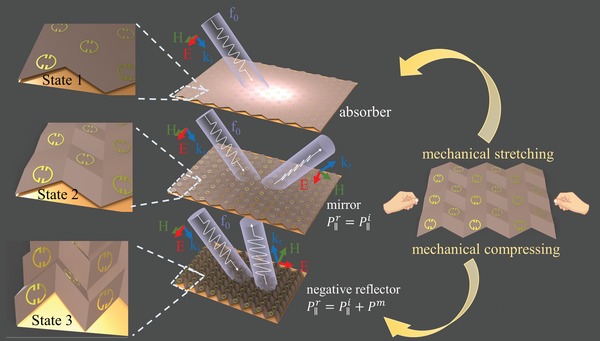
Schematic illustration of the reconfigurable metawall with a Miura‐ori pattern. Under external mechanical stimuli, the metawall could be switched freely between various deformation: absorber at the unfolded geometry (state 1), mirror (state 2) at minor folding angles, and polarization‐conversion negative reflector (state 3) at large folding angles. Mirror does not change the incident momentum ($P_{∥}^{i}$), while negative reflector provides a reversed momentum (*P^m^*) to the incident one and results in negative reflection.

The schematic illustration of the proposed origami‐based metawall is shown in Figure 1. First, periodical arrays of copper split‐ring resonators offering strong magnetic response are printed on a flat sheet to construct a 2D metasurface. The optical responses of the metawall depend on the interactions of neighboring meta‐atoms, determined by their relative 3D attitudes as well as the lattice constant of the Miura‐ori.

The misaligned meta‐atoms within the folded metawall, on the one hand make the interactions more complicated, on the other hand provide an additional degree of freedom to manipulate light. In order to guarantee high‐performance of the metawall at folded state and simultaneously simplify the process of structural optimization, each unit cell is composed of double split‐ring resonators.^37^, ^45^ The metawall is located on top of a copper layer to guarantee zero transmission. Notably, the distance between the bottom of the metawall and copper layer remains constant over the whole transformation process of the metawall. Periodical magnetic currents oriented along *z* direction can be excited by obliquely incident waves and most of the incident power can be absorbed at the resonance mode of split‐ring resonators. Next, the 2D metawall is transformed into 3D geometries following the folding principle of the Miura‐ori pattern.^46^ The Miura‐ori unit in our design is composed of four identical parallelograms that are connected by convex mountain and concave valley creases. Vertices are formed when four creases intersect and each parallelogram is preserved as a rigid facet in the folding process. In this context, we assume the Miura‐ori pattern is folded from an ideal material with infinite stretching modulus and therefore it has only one degree of freedom described by the folding angle θ (see Figure S1, Supporting Information). The folded metawall flips the normal momentum of incident light, as an ordinary mirror behaves. Furthermore, the metawall with a slight deformation for instance, θ = 5°, does not change the in‐plane momentum of incident light while large deformation (e.g., θ = 60°) provides extra exchanges of the in‐plane momentum (*P^m^*) between light and the metawall, so as to achieve negative reflection.

The underlying mechanism for optical transition of the metawall from energy absorber to momentum deflector is illustrated in **Figure** **2**. Split ring resonators are modeled as magnetic currents under various folding states, due to their magnetic dipolar responses. As shown in Figure 2a,b, red and blue arrows represent the magnetic currents of the first and second dipoles in a unit cell, the notation $I_{mi}^{x,y,z}$ represents the magnetic current component on the first (*i* = 1) and second (*i* = 2) dipoles respectively. The periodicity along the *x* direction is designed to keep only (−1)0 and 00 propagating Floquet modes, where the first and second indices are the Floquet orders with respect to the *x* and *y* axes, respectively. For the sake of simplicity, we assume no radiation in *y–z* plane. For the 2D metawall, the induced magnetic currents are oriented along *z* direction (see Figure 2a). According to the Floquet modal analysis,^47^ the radiated fields for *n*0 Floquet mode is transverse electric (TE) wave with electric filed written as

(1) $$E_{n0}=\;y\left(\Phi_{n0}\;+\;\Psi_{n0}\right)\exp\left(ik_{xn0}x\;+\;ik_{zn0}z\right)$$

**Figure 2:**
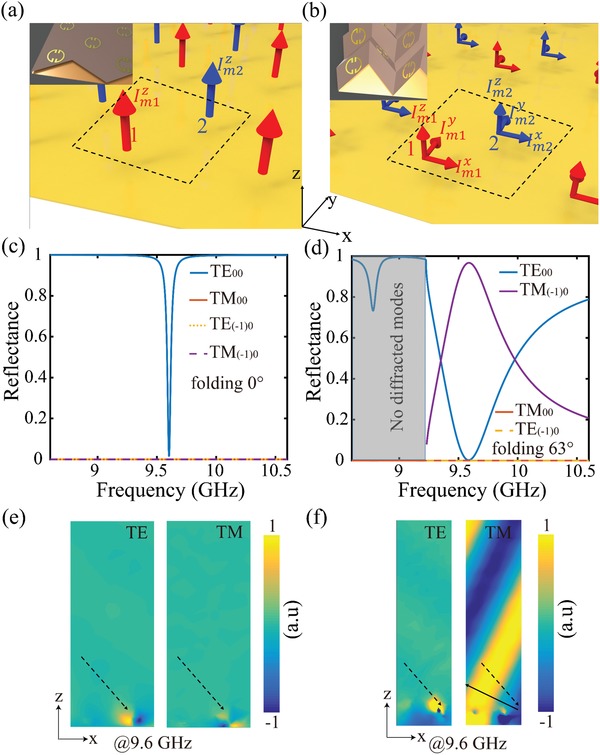
Underlying mechanism of the optical transition in the metawall. Demonstration of the induced magnetic currents located over a ground plane for a) 2D metawall and b) 3D metawall. Red and blue arrows represent the induced magnetic currents of the first and second dipoles. The inset show the 2D planar and 3D folded origami metawall. Reflectance of propagating modes versus frequency for c) 2D metawall and d) 3D metawall folded into 63°. The corresponding reflected electric field distribution are plotted in (e) and (f). Dashed arrow in each panel represents incident TE plane wave with oblique incident angle θ_in_ = 40°, while solid arrow in (f) represents the reflected TM plane wave with reflection angle *θ_r_* = −69.5°.

For *n*0 Floquet mode, $k_{xn0}=\;k_{x0}+\frac{2n\pi }{b}\;=\;k_{0}\sin\theta_{n0}$ is the wave number in the *x* direction, $k_{zn0}=\sqrt{k_{0}^{2}-k_{xn0}^{2}}=k_{0}\cos\theta_{n0}$ is the wave number in the *z* direction, the term *Φ_n0_* (*Ψ_n0_*) is expression related to magnetic currents on the first (second) dipoles, the distance between copper layer and split ring resonators, and the lattice constant of the Miura‐ori.^47^, ^48^, ^49^ Assuming the metawall is illuminated with obliquely incident TE waves propagating in the *z–x* plane, the electric field of direct reflected wave from the ground plane is expressed as

(2) $$E_{r}\;=\;yE_{0}\exp\left(ik_{x00}x\;+\;ik_{z00}z\right)$$

where *E*_0_ is the amplitude of incident wave. In order to realize perfect absorption at planar state, we need to make sure that the direct reflection from the ground plane can be cancelled by the 00 Floquet mode and all higher‐order fields dissipate through near field interactions, leading to the condition

(3) $$\begin{array}{l} \Phi_{00}+\Psi_{00}+E_{0}=0\\ \Phi_{\left(-1\right)0}+\Psi_{\left(-1\right)0}=0 \end{array}$$

This requirement can be achieved through structural optimization. With mechanical compression, the deformation of the metawall along the third dimension induces extra transverse magnetic currents on each dipole as illustrated in Figure 2b. The interactions of the neighboring transverse magnetic currents (that is, magnetic currents along both *x* and *y*‐directions) are essential for redirecting the incident wave toward an anomalous direction with polarization conversion, which is demonstrated theoretically and experimentally in previous work.^37^ The radiation fields for *z‐* and *x*‐oriented magnetic currents are TE mode while *y*‐oriented magnetic current is transverse magnetic (TM) mode. The radiated field in terms of TE plane wave can be expressed as

(4) $$E_{n0}^{TE}\;=\;y\left(\Phi_{n0}^{TE}\;+\;\Psi_{n0}^{TE}\right)\exp\left(ik_{xn0}x\;+\;ik_{zn0}z\right)$$

The term $\Phi_{n0}^{\mathrm{TE}}(\Psi_{n0}^{\mathrm{TE}})$ is expression related to *z‐* and *x*‐oriented magnetic currents on the first (second) dipoles, the distance between copper layer and split ring resonators, and the lattice constant of the Miura‐ori.^37^, ^48^, ^49^

The radiated fields in terms of TM plane waves can be expressed as

(5) $$E_{n0}^{TM}=\left(x\;-\;z\frac{k_{xn0}}{k_{zn0}}\right)\left(\Phi_{n0}^{TM}+\Psi_{n0}^{TM}\right)exp\left(ik_{xn0}x\;+\;ik_{zn0}z\right)$$

The term $\Phi_{n0}^{TM}(\Psi_{n0}^{TM})$ is induced by the y‐oriented magnetic currents on the first (second) dipole like wisely.

Since the folded metawall adds an extra momentum *P^m^* to incident in‐plane momentum $P_{\left|\right|}^{i}$, the reflection momentum differs from the incident one and anomalous reflection occurs. In order to fully transmit the incident wave to an anomalous direction, both the co‐ and cross‐polarized wave into the specular channel have to be suppressed totally. The copolarized energy for specular direction can be cancelled by the direct reflection from the ground plane, and the cross‐polarized one can be cancelled through near field interactions between two dipoles, leading to the condition

(6) $$\begin{array}{l} \Phi_{00}^{TE}+\Psi_{00}^{TE}+E_{0}=0\\ \Phi_{00}^{TM}+\Psi_{00}^{TM}=0 \end{array}$$

Based on Equation (6), the metasurfaces will redirect the incident light into an anomalous direction with polarization conversion satisfying

(7) $$\Phi_{-\left(1\right)0}^{TE}+\Psi_{-\left(1\right)0}^{TE}\;=\;0$$

which depicts the folded metawall here and previous case,^37^ and without polarization conversion satisfying

(8) $$\Phi_{-\left(1\right)0}^{TM}+\Psi_{-\left(1\right)0}^{TM}\;=\;0$$

which is the similar case described previously.^48^

As a proof of concept, an origami metawall has been designed in the microwave region with its geometric parameters included in the Supporting Information. Since the Miura‐ori pattern has a negative Poisson's ratio, both the *x*‐ and *y*‐direction periodicities of the unit cell decrease as the folding angle increases. The bottom of the metawall is rested on a foam sheet with a tiny thickness *h* (1 mm), located on top of a copper layer. To demonstrate the dynamical control over the energy and momentum of incident light, the metawall is illuminated with TE plane waves propagating in the *z–x* plane with obliquely incident angle θ_in_ = 40°. The simulated results were calculated by using commercial software (CST Microwave Studio). Periodic boundary conditions were employed along the *x* and *y* axes. For the *z*‐direction, perfectly matched layer boundary is applied. To simplify the simulation process for folded structure, we replaced each parallelogram substrate by a circle pad (with a permittivity of 3.5) with an identical radius of the split‐ring resonator. This simplification slightly shifts the resonant frequency and the underlying physics of metawall will not be affected.^38^ The reflectance of different modes is plotted in Figure 2c,d for the metawall under the unfolded state and the folded state of θ = 63° respectively. At the operating frequency *f*_0_ = 9.6 GHz, the energy carried by incident light is largely absorbed by the 2D metawall, whereas the metawall with large deformation adds an extra momentum to the incident one, leading to negative reflection at −69.5° with polarization conversion. The reflected electric fields show the consistent behavior in Figure 2e,f.

**Figure** **3**a,b shows the reflection and absorption versus the folding state at the operating frequency *f_0_* = 9.6 GHz. It indicates a high‐performance absorption with efficiency higher than 98% for the 2D metawall and a dramatic decrease occurs when the metawall is deformed into 3D geometries. The bandwidth of absorption spectrum is extremely narrow and a slight deformation of the metawall shifts the frequency of absorption, leading to this abrupt change (see Figure S2, Supporting Information). When the structure is mechanically compressed with a slight deformation, i.e., folding angle θ = 10°, 96% of the incident power could be transmitted toward the TE_00_ mode, and 1.9% toward TM_(−1)0_ mode. At this folding state, the surface currents on each split ring resonator are negligible (not shown here). Therefore, the structure behaves like a reflective mirror. With continuous deformation, the TE_00_ decreases and TM_(−1)0_ increases gradually. The reflected power into TM_(−1)0_ reaches its maximum value 98.3%, while TE_00_ is suppressed totally with the folding angle θ = 63°. Over the whole folding procedure, TM_00_ and TE_(−1)0_ are both suppressed completely (see Figure S2, Supporting Information). Considering the practical application scenario, the angular performance of the metawall is further investigated as shown in Figure S3 (Supporting Information). The incident angle mainly influences the efficiency of the metawall in absorption and negative reflection domains, and notably the metawall show good tolerance to the deviation of incident angle which will guarantee its good performance in practical applications. Besides, with the incident angle increases, the transition from reflective mirror to negative reflector occurs at larger folded state. To demonstrate the proposed origami metawall, a sample truncated to finite size of 9 × 8 unit cells in the *x‐* and *y*‐directions, respectively, have been fabricated and characterized in the microwave region. The fabricated sample is located on top of a copper layer, separated by a piece of foam with the relative permittivity of 1.1 and the thickness of 1 mm. Figure 3a,b shows the measured reflection amplitude and absorption versus folding angle. Compared with the simulation results, the working frequency of the fabricated sample undergoes a slight, shifting from 9.6 to 9.24 GHz (see measured S parameter in Figure S3a, Supporting Information). Neglected parts of the parallelogram substrate and fabrication errors attribute to this slight redshift. The measured absorption is higher than that from numerical calculation at folded cases, as a result of irregular scattering introduced by the finite imperfect fabricated sample.

**Figure 3:**
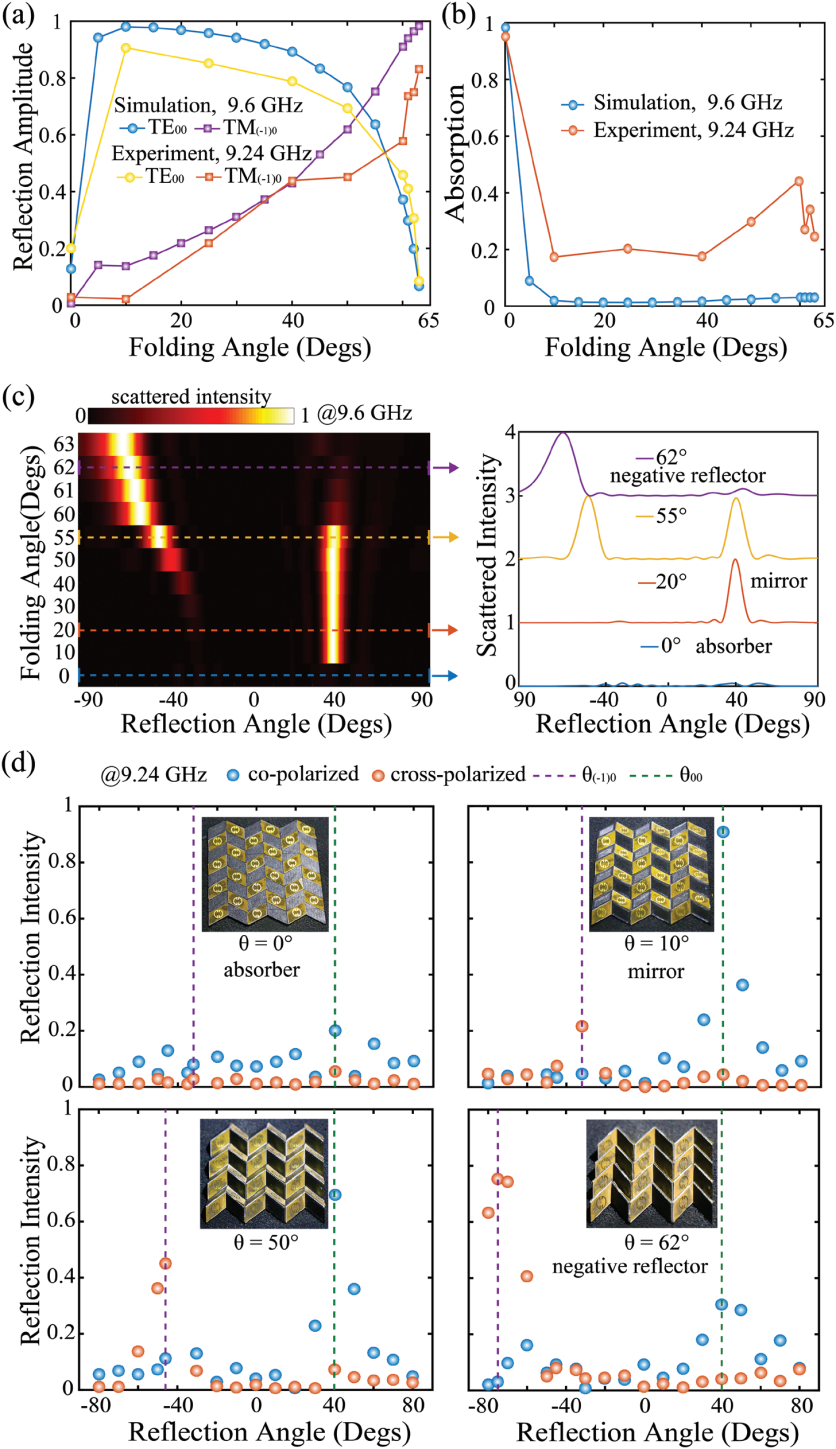
Function of metawall transits from an absorber, a mirror, to a negative reflector. a) Reflection amplitudes of TE_00_ and TM_(−1)0_ under various folding angles. b) Absorption of the metawall versus folding angles. c) Simulated results of the scattered intensity versus reflection angle at different folding states. Right panel shows the scattering spectra with the folding angle at *θ =* 0°, 20°, 55°, and 62°. d) Measured reflection intensity versus reflection angle with the metawall tuned to 0°, 10°, 50°, and 62°, respectively.

We proceed to investigate the scattering properties of the origami metawall at various folding angles. Figure 3c shows the simulated scattered intensity of a finite size with 9 × 8 unit cells. To obtain a measurement of the absorption efficiency, the scattered intensity is normalized to the specular reflection of a perfect electric conductor with the same size of the unfolded metawall, while the scattered intensity is normalized to its own maximum for folded structures to better present the scattering direction. As the folding angle increases from 10° to 50°, the intensity of the specular reflection mode at the reflection angle of 40° is almost unchanged, which means the specular reflection is dominant at these states. With the increasing of folding angle from 55° to 63°, the intensity of the specular reflection mode vanishes suggesting negative reflection is dominant at these domains. The specular reflection angle is equal to the incident one, while the negative reflection angle for (−1)0 mode can be express as

(9) $$\theta_{\left(-1\right)0}=\;arcsin\left(sin\theta_{\mathrm{in}}-\;\frac{\lambda }{p}\right)$$

where θ_in_ is the incident angle, λ is the working wavelength, and *p* is the periodicity along the *x‐*direction. The periodicity of the metamaterial decreases as the folding angle increases, thus the scattered direction of (−1)0 mode deviates more and more from the normal direction. Figure 3d shows the measured reflection characteristics of the fabricated metawall with various folding angles at 0°, 10°, 50°, and 62° (the result of 25° and 63° are shown in Figure S6, Supporting Information). The purple dotted line represents the reflection angle for (−1)0 mode, calculated as −31.9°, −32.2°, −46.1°, and −74.8°, respectively. The brown dotted line represents the specular reflection angle θ_00_ = 40°, while the symbols of blue and orange balls represent the measured reflection intensity for co‐ and cross‐polarized respectively. All the measured intensities are normalized by the specular reflection intensity of a metallic plate under incident angle θ_in_ = 40°.

Angular dispersion—the response of a metasurface strongly depending on the impinging angle—is an intrinsic property of metasurfaces.^50^ In some circumstances, such fundamental effects in metasurfaces will limit their practical applications. An intimately known scenario is incident angle‐dependent wave‐front control via phase‐gradient metasurfaces. The in‐plane momentum added by negative reflector made of phase‐gradient metasurfaces is proportional to the gradient of the local reflection phase and has negligible dependence on the incident angle.^51^ Consequently, the reflection angle varies as the incident one changes as illustrated in **Figure** **4**a. The geometry transformation in origami is exactly a promising approach to realize incident angle‐independent wave‐front control, that is, efficient compensation on the intrinsic angular dispersion in gradient metasurfaces. The reason is its ability of continuous deformation on periodicity, which is the main challenge of angular sensitivity in ordinary gradient metasurfaces or metagratings. Compared with foldable 1D grating structure (which could be periodic metallic stripes printed on a polyimide substrate), the Miura‐ori pattern is more suitable for engineering deployable or foldable structures due to its high degree of symmetry embodied in its periodicity, and three important geometric properties which is beyond the limitations of 1D foldable grating structure: it can be rigidly folded (that is, it can be continuously and isometrically deformed from its flat, planar state to a folded state); it has only one isometric degree of freedom, with the shape of the entire structure determined by the folding angle of any single crease; it exhibits negative Poisson's ratio (folding the Miura‐ori decreases its projected extent in both planar directions).^44^

**Figure 4:**
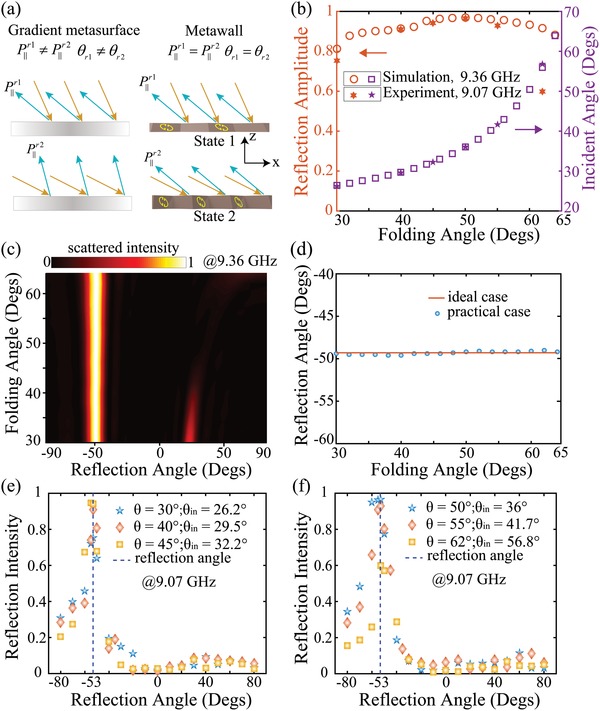
Negative reflection at a fixed reflection angle. a) Schematic illustration of reflection upon a gradient metasurface and the proposed metawall. The gradient metasurface adds a constant momentum to the incident light thus the reflection momentum ($P_{∥}^{r}$) varies with incident angle, whereas the metawall provides adaptive momentum to guarantee the reflection momentum is independent on the incident angle and negative reflection at a fixed outgoing angle is realized. b) Reflection amplitude and incident angle versus the folding state. In simulations, the reflection angle is fixed at −49.3° and this value is revised to −53° in experiments for the sake of slight redshift of the operating frequency. c) Simulated scattering properties under different incident angles. d) Reflection angle for ideal and practical cases versus folding state. Measured reflection intensity versus reflection directions with incident angle at: e) 26.2°, 29.5°, 32.2°, and f) 36°, 41.7°, and 56.8°.

The resonance frequency of the metawall undergoes a slight shift with the increasing of folding angle from 30° to 60° (see Figure S2, Supporting Information). We define such phenomenon “state dispersion”—the working frequency depends on the state of the metawall. On the other hand, different incident angles will shift the resonance frequency of the metawall as well, this is an intimately known scenario “angular dispersion.” Here state dispersion and angular dispersion cancel out each other, providing a high‐performance negative reflection at a fixed outgoing angle under a continuous range of incident angles from 26.4° to 63.9° while the folding state ranges from 30° to 60°. To demonstrate this behavior, the metawall is illuminated with 9.36 GHz TE plane waves under a continuous range of incident angles from 26.4° to 63.9°. For a known incident light, the metawall is tuned to a required folding state by mechanically compressing or stretching and negative reflection at the reflection angle θ_r_ = −49.3° is realized. Figure 4b shows the relation between incident angle and the folding state. The high efficiency of the metawall is validated by the reflection amplitude as well. Figure 4c shows the scattered intensity of a finite size with 9 × 8 unit cells under various folding angle. It is obvious that the specular reflection has been suppressed largely and the incident light is redirected into the vicinity of θ_r_ = −49.3°. To clarify this more clearly, Figure 4d further plots the reflection angle under different folding state. The orange solid line represents θ_r_ = −49.3° for ideal case while the symbols of the blue balls represent the practical case with a finite size. Compared with the simulation results, the working frequency of the fabricated sample undergoes a slight, redshift from 9.36 to 9.07 GHz (see measured S parameter in Figure S4b, Supporting Information). For practical operating frequency *f*_1_ = 9.07 GHz, the negative reflection angle θ_(−1)0_ is corrected to −53°. Figure 4e,f shows the measured reflection intensity versus reflection angle under various incident angles. The blue line in each figure shows the corrected negative reflection angle where the incident waves are reflected back exactly to the same directions. In all cases, the measured intensity is normalized by the specular reflection from the metallic plate under oblique incident angle at 36° (for this incident angle, the metawall is folded at 50°).

In this work, we propose and demonstrate a reconfigurable origami metawall for dynamical control over the energy and momentum transfer of light simultaneously through mechanical deformation of the Miura‐ori pattern. The deformation of the metawall into the vertical direction sustains transverse magnetic currents that do not exist in the unfolded metawall, which contributes to the distinctive manipulations of light. Furthermore, the continuously geometric deformation of the Miura‐ori lattice makes the metawall independent on the incident angle for wave‐front control, that is, efficient compensation on the intrinsic angular dispersion in gradient metasurfaces. Adaptively negative reflection at a fixed outgoing angle is available by simply adjusting the folding angle. With the aid of the 3D printing technology, a proof‐of‐concept reconfigurable metawall with Miura‐ori pattern is constructed and the electromagnetic transition performance is validated experimentally at microwave frequencies. The present origami metawall may open an avenue toward lightweight and deployable metadevices with customized diversified and high‐contrast electromagnetic properties.

## Experimental Section

*Device Fabrication*: The copper split‐ring resonators (thickness 0.035 mm) were fabricated by a standard printing circuit board technology, periodically printed on a Halogen‐free frame‐resistant type polyimide film (thickness 0.05 mm, permittivity 3.5). The Miura‐ori substrate (thickness 0.3 mm) was fabricated by 3D printing with a permittivity of 2.5 printed with a 3D printing technology (Ultimaker 2 extended+). Polylactic acid was selected as the printing material. The ultrathin film with copper split‐ring resonators was then stuck on the Miura‐ori substrate. Notably, the thickness of the dielectric substrate at the creases was smaller than other places of the parallelogram; such configuration had a great flexibility to alter its folding state mechanically (see the inset in Figure 2d).

*Measurement*: The optical transition of the fabricated origami metawall was experimentally verified in an anechoic chamber with a setup of two wideband double‐ridged horn antennas connected with a vector network analyzer. The origami metawall composing of 12 × 13 unit cells was put at the center of an arc track with a radius of 1.2 m. The two horn antennas were mounted on the arc track. One of the horn antennas served as the source to illuminate oblique incidence plane wave to the origami metawall. TE and TM‐polarized waves could be generated by rotating the antenna. Another horn antenna worked as the receiver moving along the arc track to measure the co‐ and cross‐polarized scattered fields at desired angles.

## Conflict of Interest

The authors declare no conflict of interest.

## Supporting information

Supporting Information
