# Supplementary material for: Origami Metawall: Mechanically Controlled Absorption and Deflection of Light
Source: Adv Sci (Weinh). 2019 Oct 29;6(23):1901434. doi: 10.1002/advs.201901434 (PMC6891917; doi:10.1002/advs.201901434)
Supplement: Supplementary file 1 — Supporting Information [file ADVS-6-1901434-s001.pdf]

Copyright WILEY-VCH Verlag GmbH & Co. KGaA, 69469 Weinheim, Germany, 2019.

ADVANCED SCIENCE
Open Access

Supporting Information

for *Adv. Sci.,* DOI: 10.1002/advs.201901434

Origami Metawall: Mechanically Controlled Absorption and Deflection of Light

Min Li, Lian Shen, Liqiao Jing, Su Xu, Bin Zheng, Xiao Lin, Yihao Yang, Zuojia Wang, and Hongsheng Chen**

Supporting Information

Title: **Origami Metawall: Mechanically Controlled Absorption and Deflection of Light**

Min Li[1,2#], Lian Shen[1,2#], Liqiao Jing[1,2,3], Su Xu[5], Bin Zheng[1,2], Xiao Lin[6], Yihao Yang[6], Zuojia Wang[4,], and Hongsheng Chen[1,2*]*

1. Geometry of the metawall with Miura-ori pattern.

The geometry of a unit cell of the designed metawall with Miura-ori pattern is plotted in **Figure S1**. The parameters of the parallelogram are fixed as a = 10 mm, b = 16 mm and γ = 60°. Two split-ring resonators are located at the centers of the diagonal parallelograms with identical dimensions. The inner and outer radii of each split-ring resonator are r_1 = 2.8 mm and r_2 = 3.3 mm, respectively, while the gap size is g = 0.7 mm and s = 2.7 mm. The solid (dashed) lines correspond to the mountain (valley) creases. Vertices are formed when four creases intersect and each parallelogram is preserved as a rigid facet in the folding process. In this context, we assume the Miura-ori pattern is folded from an ideal material with infinite stretching modulus and therefore it has only one degree of freedom described by the folding angle θ.

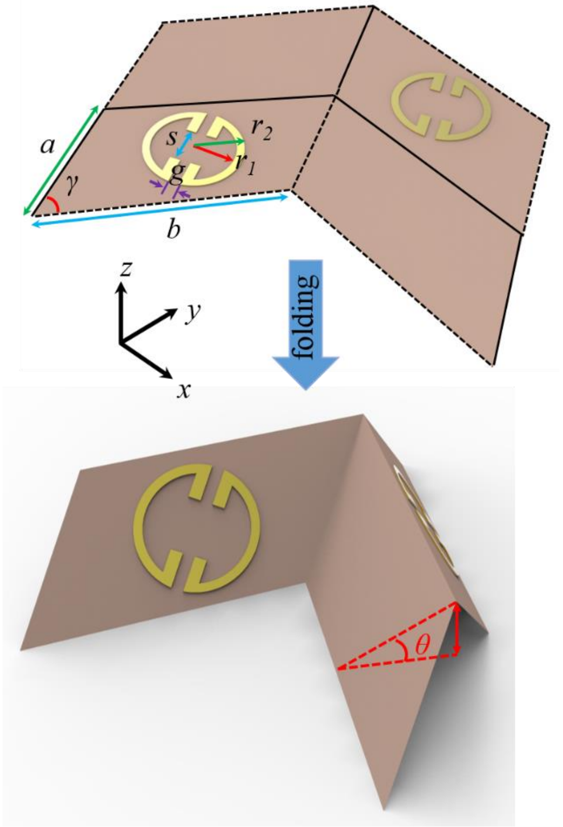

Figure S1. The geometry of a unit cell of the metawall with Miura-ori pattern.

2. Reflectance spectrum of all propagating modes

Figure S2 shows the simulation results of the reflectance versus frequency under various folding state. The origami metawall is illuminated with TE plane wave propagating in *z*-*x* plane under obliquely incident angle θ_{in} = 40°. The horizontal line in each panel represents the reflectance under various folding state for f_0 = 9.6 GHz. Over the whole deformation, TM_{00} and $TE_{(-1)0}$ is highly suppressed, guaranteeing the efficiency of the metawall. For unfolded 2D metawall, the bandwidth of absorption spectrum (see TE_{00} spectrum) is extremely narrow and a slight deformation of the metawall shifts the frequency of absorption, leading to a dramatic decrease of absorption efficiency, which is shown in **Figure 2b**.

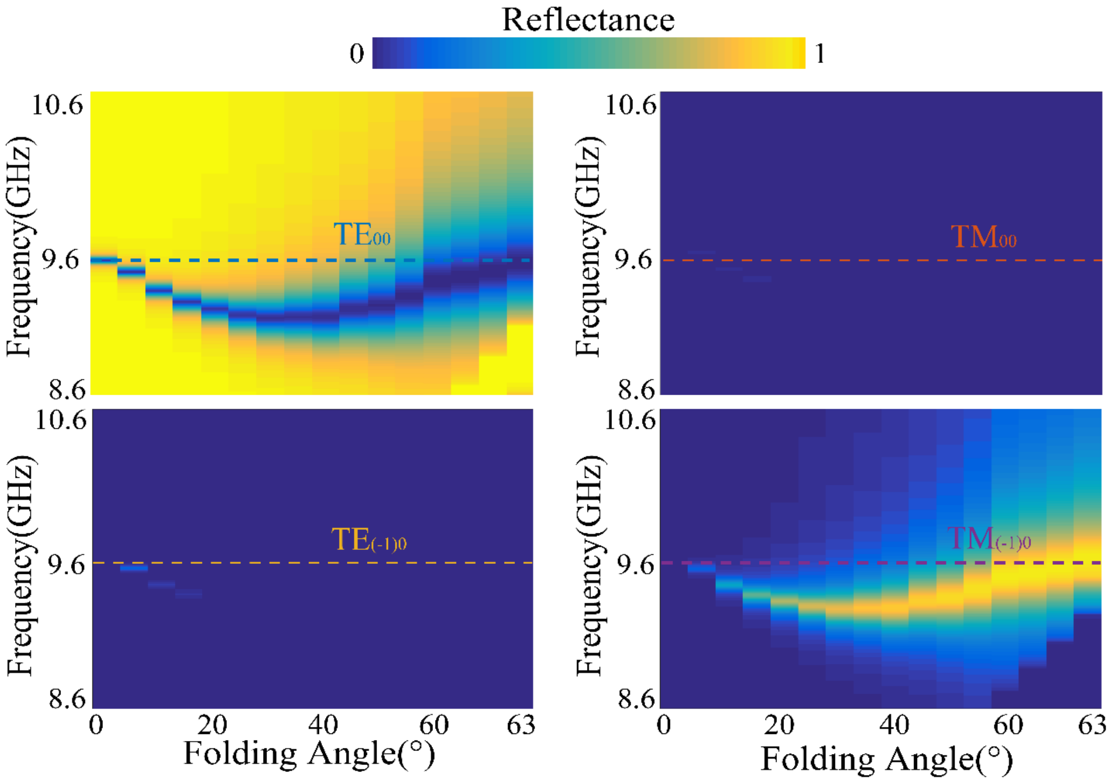

Figure S2. Simulated reflectance versus frequency for different propagating Floquet modes under various folding angles. The horizontal line on each figure shows the reflectance for f_0 = 9.6 GHz.

3. Angular performance of the metawall in three states.

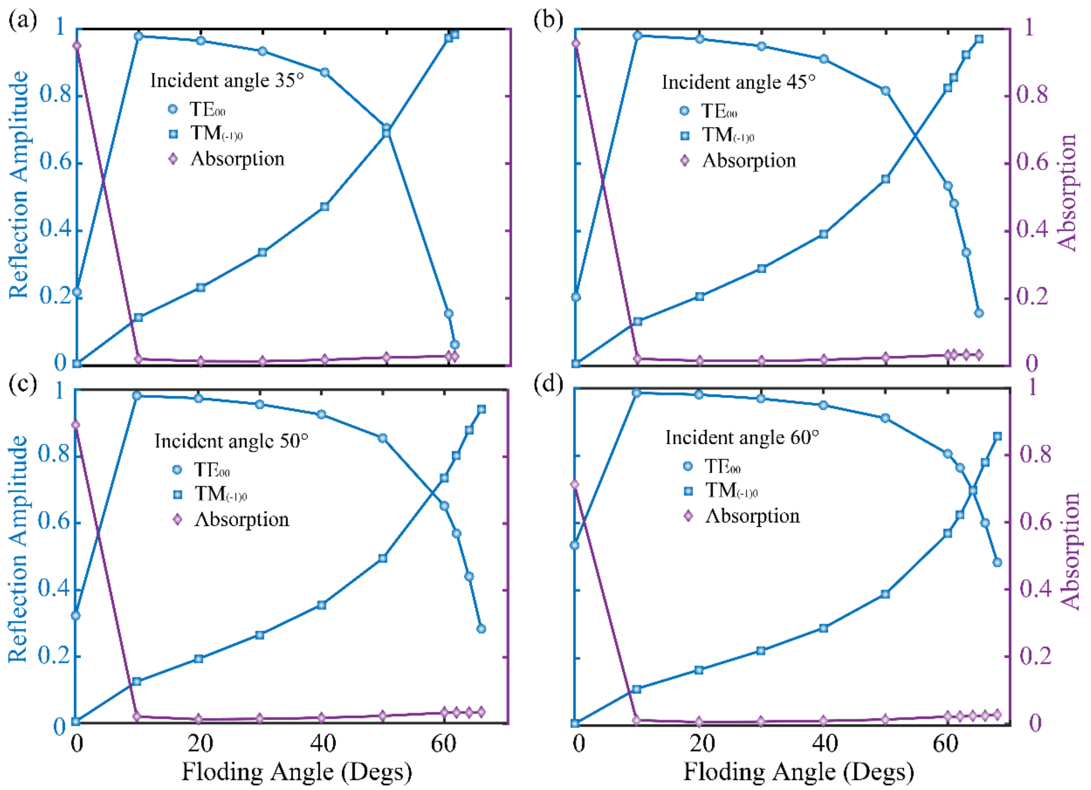

Figure S3. Function transitions of the metawall, from an absorber, a mirror, to a negative reflector, under incident angle a) 35°, b) 45°, c) 50°, d) 60°.

4. Redshift of working frequency for the fabricated metawall.

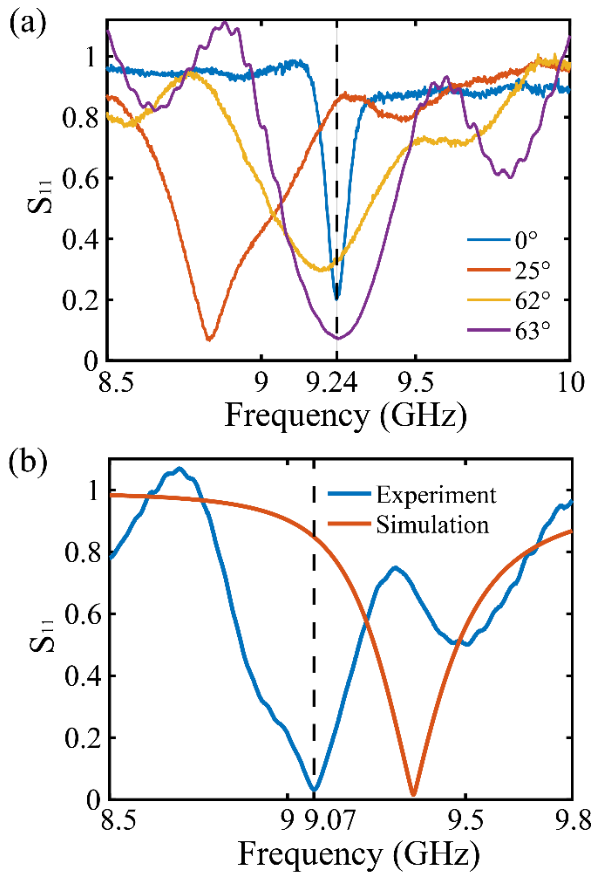

Figure S4. a) Illuminated with obliquely incident angle at 40°, measured S parameter under various folding angles. b) Illuminated with oblique incident angle at 36° measured and simulated results of S parameter when the metawall is folded to 50°.

To investigate the optical transition of the fabricated metawall, we measured S_{11} parameter under different folding state with oblique incident angle at 40°, as shown in **Figure S4a**. Compared with simulation results shown in **Figure S2,** the working frequency undergoes a redshift from 9.6 GHz to 9.24 GHz. Neglect parts of the parallelogram substrate and fabrication errors attribute to this slight redshift. Another fascinating application of metawall is to realized negative reflection at fixed reflection angle. To obtain a high efficiency of negative reflection under different incident angles, the folding angle of metawall is optimized to 50° and the corresponding

incident angle is 36°. According to Equation (9) in main text, the reflection angle is calculated as -49.3°. For different incident angles, the metawall can be tuned to a certain folding state to keep the reflection angle unchanged. As illustrated in **Figure S4b**, the working frequency of the fabricated metawall undergoes a slight, redshift from 9.36 GHz to 9.07 GHz. For practical operating frequency f_1 = 9.07 GHz, the negative reflection angle $\theta_{(-1)0}$ is corrected to -53°.

5. Required conditions to suppress higher order modes

Illuminated with TE plane waves propagating in *z-x* plane, the periodicity along the *y* direction is smaller than the wavelength to guarantee no radiation in the *z-y* plane; to ensure sure (-1)0 and 00 are the only possible propagating diffraction orders, the closest higher-order modes [i.e., 10 and (-2)0] are evanescent and the required conditions to suppress higher-order modes are given by [1]:

$$\begin{aligned} &\theta_{(-1)0} < arcsin(2\,sin\,\theta_{in} - 1), 0 < \theta_{in} < arcsin(\frac{1}{3}) \\ &\theta_{(-1)0} < arcsin(\frac{1}{2}sin\,\theta_{in} - \frac{1}{2}), arcsin(\frac{1}{3}) < \theta_{in} < \frac{\pi}{2} \end{aligned} \quad \text{S(1)}$$

where $\theta_{(-1)0}$ and θ_{in} are the reflection angle for (-1)0 Floquet mode and incident angle respectively. It can be shown that knowing the incidence angle θ_{in}, the metasurface period p and the wavelength λ, the diffraction angle for (-1)0 mode is fixed as:

$$\theta_{(-1)0} = arcsin(\,sin\,\theta_{\text{in}} - \frac{\lambda}{p}) \quad \text{S(2)}$$

Take the circumstance for investigating optical transition of the metawall as an example, i.e., θ_{in} = 40°. The diffraction angle for (-1)0 mode is plotted in **Figure S5**.

Blue line represents the calculated results of the maximum value of $\theta_{(-1)0}$ = -10.3°. Orange and yellow lines show simulated and experimental results of $\theta_{(-1)0}$ respectively. The operating frequency of the practical fabricated metawall undergoes a redshift, leading the experimental $\theta_{(-1)0}$ smaller than the simulated one. Both the simulation and experiment results are smaller than the calculated maximum value, guaranteeing (-1)0 and 00 are propagating modes while higher order modes been evanescent.

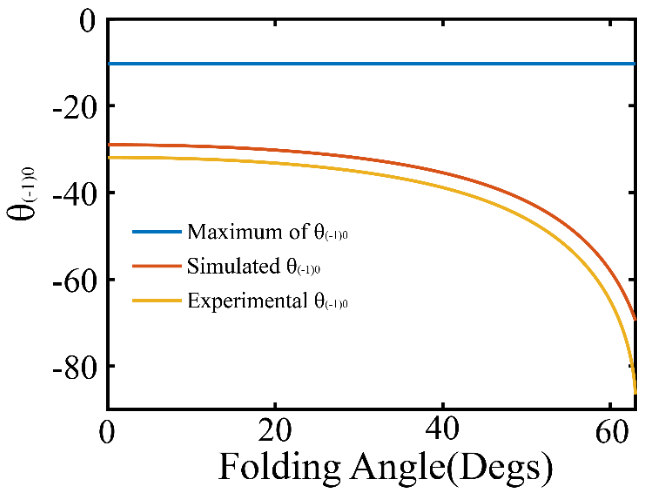

Figure S5. Reflection angle of (-1)0 Floquet mode versus folding angle θ. Over the whole range of deformation, both the simulation and experiment results are smaller than the calculated maximum value, guaranteeing (-1)0 and 00 are propagating modes while higher order modes been evanescent.

6.

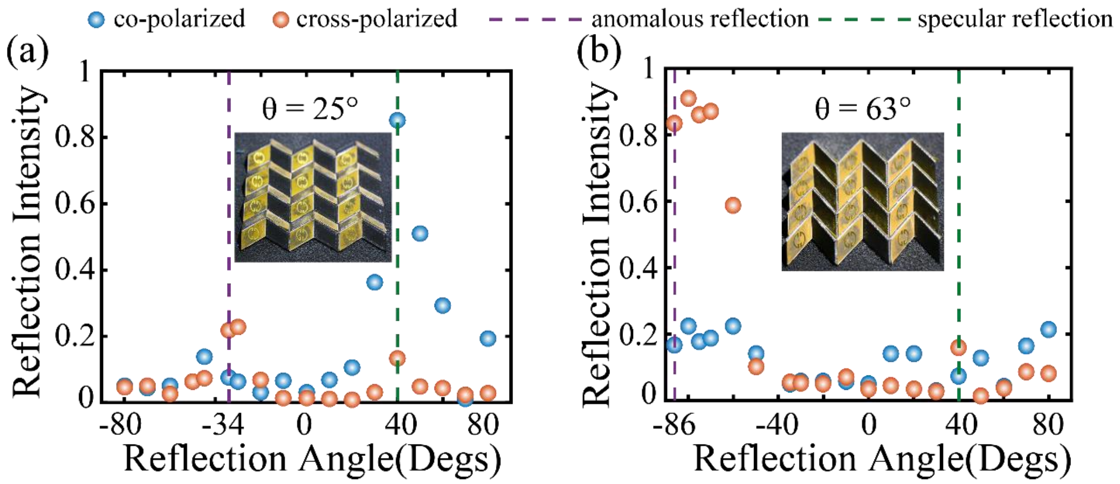

Figure S6. Measured reflection intensity versus reflection angle with folding angle at: a) 25° and b) 63°. The purple dotted line represents the reflection angle of $\theta_{(-1)0}$. The brown dotted line represents the specular reflection angle θ_{00} = 40°, while the symbols of blue and red balls represent the measured reflection intensity for co and cross-polarized respectively.

References:

[1] Y. Ra'di, D. L. Sounas, A. Alù, *Phys. Rev. Lett.* **2017**, *119*, 67404.
